# Direct microscopic appearance of *Acinetobacter baumannii* in nosocomial meningitis after transsphenoidal surgery

**DOI:** 10.11604/pamj.2026.53.54.51251

**Published:** 2026-02-04

**Authors:** Didi Mehdi, Khallikane Said

**Affiliations:** 1 Intensive Care Unit, University of Caddy Ayad, Military Hospital of Avicenne, Marrakech, Morocco

**Keywords:** Nosocomial meningitis, *Acinetobacter baumannii*, direct microscopic

## Image in medicine

A 47-year-old woman underwent transsphenoidal surgery for resection of a meningioma and was admitted to the intensive care unit (ICU) for postoperative monitoring. During her intensive care unit stay, she developed a high-grade fever associated with progressive deterioration of consciousness. Lumbar puncture was performed due to suspicion of central nervous system infection. The spinal fluid was macroscopically turbid. Direct microscopic examination after Gram staining revealed numerous small Gram-negative coccobacilli predominantly arranged in diplococci form, diffusely distributed across the microscopic field (image). This pleomorphic morphology initially suggested Gram-negative diplococci but was compatible with *Acinetobacter* species. Cerebrospinal fluid culture subsequently confirmed *Acinetobacter baumannii* with a multidrug-resistant profile, susceptible only to colistin and tigecycline. Despite targeted intravenous colistin therapy and intensive supportive care, the patient’s condition rapidly deteriorated and resulted in death. Given the transnasal surgical approach and the temporal association with nasogastric tube insertion during the ICU stay, an ascending nosocomial infection was strongly suspected. This image highlights the diagnostic importance of recognizing the characteristic direct microscopic appearance of *Acinetobacter baumannii*, which may be misleading, and underscores the critical role of strict aseptic technique and infection-prevention measures during routine invasive procedures.

**Figure 1:**
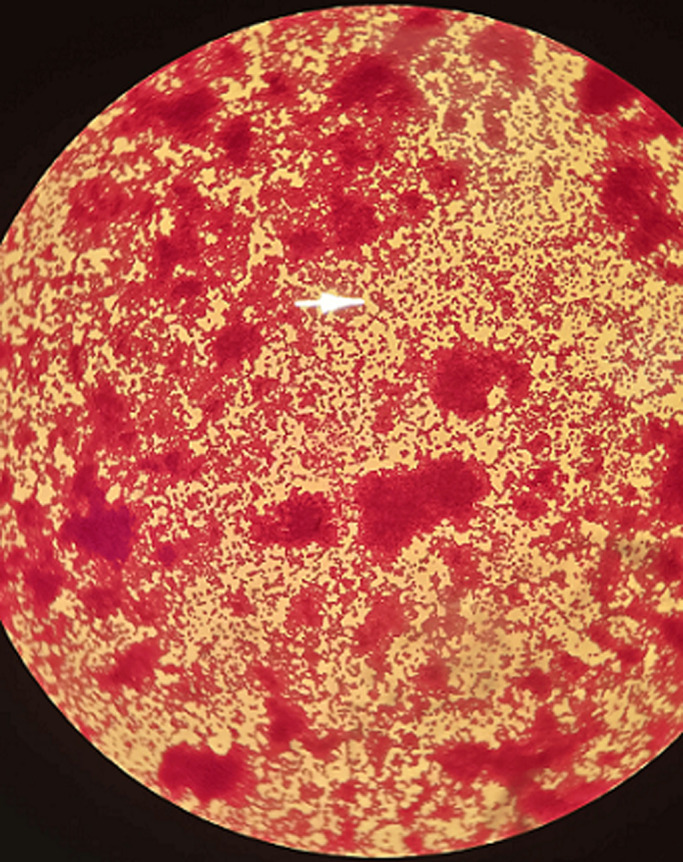
direct examination of the spinal fluid after Gram staining showing numerous small Gram-negative coccobacilli, arranged predominantly in diplococci form, diffusely distributed across the microscopic field

